# Plant provenance can influence the impacts of temperature and moisture on intraspecific competition in *Pseudoroegneria spicata*

**DOI:** 10.1002/ece3.10603

**Published:** 2023-10-24

**Authors:** Sabina Donnelly, Morodoluwa Akin‐Fajiye, Lauchlan H. Fraser

**Affiliations:** ^1^ Department of Natural Resource Sciences Thompson Rivers University Kamloops British Columbia Canada

**Keywords:** climate change, competition, drought, native grasses, *Pseudoroegneria spicata*, warming

## Abstract

Warming and changing precipitation can alter the performance of native grasses that are essential to grassland ecosystems. Native grasses may respond to changing climate by phenotypic plasticity or lose their current ranges. Establishing plant species from southern, warmer provenances may reduce the likelihood of biodiversity loss and improve restoration success in cool, northern locations that are undergoing warming. We conducted competition trials for *Pseudoroegneria spicata* (bluebunch wheatgrass), a native grass commonly found in western North American grasslands, to understand the impact of temperature and moisture on plant–plant interactions. We obtained seeds from three locations along a latitudinal gradient in North America, two in British Columbia (BC), Canada, and one in California, USA. We compared the effects of warming, changing water inputs, and competitor provenance on pairwise competitive interactions among *Pseudoroegneria spicata* plants grown from seeds obtained from the three locations. We quantified interactions using the relative interaction intensity, which has values from −1 (complete competition) to +1 (complete facilitation). Target plants from northern British Columbia, the location with the coldest summer temperature, were generally more competitively suppressed when competing with plants from California, which had the warmest summer temperature and lowest summer precipitation. Competitive suppression of target plants from northern British Columbia and southern British Columbia was more intense when competitor provenance was more geographically distant from target plant provenance. Finally, plants from northern British Columbia and southern British Columbia were more suppressed at higher temperatures, indicating some local adaptation, while plants from California were not affected by competitors, temperature, or water input. Plants grown from seeds obtained from warm and dry locations appear to be more tolerant to competition at higher temperatures, compared to plants from cooler regions. Native plant diversity and restoration success in grasslands subjected to climate change may be preserved or improved by assisted migration of seeds from warm to cooler but warming locations.

## Graphical abstract

Native bluebunch wheatgrass plants from cooler locations were competitively suppressed by plants from warmer locations. Competitive suppression of plants from cooler locations was more intense when competitors were from farther away. Restoration success under climate change may be improved by assisted migration of seeds from warm to cool locations.

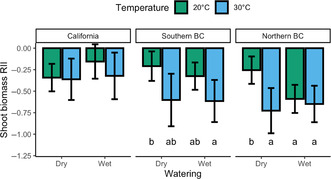

## 1 INTRODUCTION

Climate change is expected to have detrimental effects on native plants and communities (Giejsztowt et al., 2020; Upson et al., 2016). Warming and precipitation are important components of global climate change that can influence native species performance and distribution (Liu et al., 2017; Zettlemoyer et al., 2019). Native species can respond to changing climatic conditions by adapting and maintaining their current distribution, or by shifting their geographical ranges, thereby changing their distribution to stay within their climatic tolerances (Corlett & Westcott, 2013).

Phenotypic plasticity and local adaptation to different environments can guide plant species response to climate change. Phenotypic plasticity occurs when an organism displays differing phenotypes in response to changing environmental conditions (Davidson et al., 2011). While there has been much focus on the plasticity of invasive plants (Davidson et al., 2011; Richards et al., 2006), native species can also display plasticity in response to environmental change (Nicotra et al., 2010; Palacio‐López & Gianoli, 2011). Local adaptation to biotic and abiotic conditions may also lead to ecological specialization, which occurs when species display higher fitness in their native environments compared to species that do not originate from that environment (Kawecki & Ebert., 2004; Savolainen et al., 2013).

Plant species distribution may track suitable climatic conditions, thereby reducing their risk of extinction (Barnes et al., 2017; Corlett & Westcott, 2013). As temperatures warm, species may shift their distribution northward or poleward (Dolezal et al., 2016). In addition, climate change may guide plant responses to competitive interactions, as more stressful climatic conditions may strengthen intraspecific competition (Adler et al., 2018; García‐Cervigón et al., 2013). The growth of plants, collected from populations adapted to different climatic conditions, may allow us to determine how to select seeds that can respond quickly to climate change, survive intraspecific competition, and prevent mismatch between plant ecotypes and environment.

A mismatch between the phenotype and a given environment can occur when individuals become poorly adapted for the location in which they currently reside (Hendry et al., 2011; Paul et al., 2011). Assisted migration of native species has been proposed as a mechanism to mitigate the negative impacts of climate change (Vitt et al., 2010; Williams & Dumroese, 2013). Human‐assisted migration of species is the physical transfer of populations from one location, where they have previously adapted to climatic conditions, to another location that may be suitable in the future as conditions change (Gray et al., 2011). Assisted migration has been applied to conservation of extinct species (McLachlan et al., 2007; Vitt et al., 2010), but can also help preserve populations with a species, thereby maintaining native plant biodiversity in the face of climate change (Gray et al., 2011). In the context of climate change, propagules from populations adapted to a warmer climate can be transferred to an environment that is expected to become warmer. For example, in the northern hemisphere this would involve moving a genotype from the south to the north, or from low to higher elevations (Sáenz‐Romero et al., 2021). However, plant transport across latitudinal ranges may influence ecological processes, such as competitive interactions of plants responding to different abiotic conditions. Assisted migration may involve some risks (Montwé et al., 2018). For example, competition can influence the performance of populations that have been established by assisted migration. Therefore, understanding the role of competition is particularly important as we attempt to predict how climate change may influence species adaptation to new environments.

Controlled plant experiments involving pairwise species combinations have been used extensively in the study of the role of competition (Keddy & Shipley, 1989). While field experiments are ideal as they more closely mimic the environmental conditions experienced by plant species (Diamond, 1986), we conducted this experiment in the greenhouse as this allowed us to exert more control on the experimental factors and improve seed germination. Controlled experiments allow for the elimination of confounding experimental factors and the clarification of ecological theories or processes (De Roy et al., 2013).

In this study, we used intraspecific pairwise comparisons to examine the response of three populations of a native grass, *Pseudoroegneria spicata* (bluebunch wheatgrass), to aspects of climate change. While interspecific competition can influence population dynamics under assisted migration (Backus & Baskett, 2021), intraspecific competition can be equally, or even more important (Adler et al., 2018). We chose this grass because it is prevalent in several ecosystems in British Columbia (BC), Canada, and has a long latitudinal distribution down to central California in the USA (Ogle et al., 2010; Quinton et al., 1982). Bluebunch wheatgrass is a forage grass and is therefore an economically important component of rangelands in western North America (Ogle et al., 2010). We chose to examine the impact of temperature as Canada's changing climate report estimates that Canada has undergone an increase of 1.7°C between 1948 and 2016, with higher increases in northern British Columbia (Zhang, Flato, et al., 2019). Precipitation is also expected to increase in Canada, with greater increases in northern regions (Zhang, Flato, et al., 2019). These two factors are therefore important drivers of climate change in Canada and are expected to influence intraspecific competition in plants (Partzsch, 2019; Weatherford & Myster, 2011).

We tested the response of bluebunch wheatgrass seeds obtained from three locations in North America to different water and temperature treatments in the greenhouse. We addressed the following question: How do increased water inputs and elevated temperatures influence intraspecific competitive interactions between bluebunch wheatgrass plants obtained from three locations along a latitudinal gradient in western North America? We expected local adaptation to influence plant response and competition such that plant performance under experimental conditions would match conditions in their seed origin. Plants grown from seeds obtained from northern California would be competitively dominant at high temperatures, but competitively weak at low temperatures. Similarly, we expected that plants from the coldest location, that is, northern British Columbia, would be competitively dominant at low temperatures and competitively weak at high temperatures. By comparing bluebunch wheatgrass plants from different locations, we aim to understand the role of climatic conditions at source locations in the success of assisted migration of native grasses.

## 2 METHODS

### 2.1 Study species

*Pseudoroegneria spicata*, bluebunch wheatgrass, is a perennial bunchgrass species, commonly found in British Columbia, and an important forage species for wildlife and livestock. The species' distribution extends northward to Alaska, eastward to Manitoba, and southward to Texas, New Mexico, Arizona, and northeastern California (Klinkenberg, 2022; Zlatnik, 1999). The extensive distribution of bluebunch wheatgrass is perhaps in part due to its variable and adaptable traits (Larson et al., 2004). This species can range in height from 45 to 122 cm and produce spike inflorescences that vary from 7 to 20 cm in length (Ogle et al., 2010). Bluebunch wheatgrasses consist of two subspecies, ssp. *spicata* and ssp. *inermis*, in which the lemmas are awned and unawned, respectively (Klinkenberg, 2022; Ogle et al., 2010). Absence or presence of awns is caused by a single gene and is believed to necessitate separation into two species (Barkworth, 2006). For this study, seeds were collected only from the *spicata* subspecies to maintain consistency.

This study used seeds of *Pseudoroegneria spicata* obtained from three locations extending from northern British Columbia in Canada to northern California in the USA (Table 1). Sample location was based on information gathered from herbarium data, literature, and information collected from personal correspondence with professionals. Appropriate sites had healthy, naturally occurring, and contiguous populations. Collections were taken from populations that had an elevation of roughly 1000 m to control for elevation as a confounding variable. At each location, seeds were collected from individual plants at least five meters apart. Maintaining distance between individuals minimized relatedness within the population and increased the variation present within our collected seeds.

**TABLE 1:** Collection locations of *Pseudoroegneria spicata* seeds across British Columbia and California.

Location ID	Province/state (country)	Latitude/longitude	Mean annual temperature (°C)	Annual precipitation (mm)	Summer precipitation (mm)	Mean cold month temperature (°C)	Mean warm month temperature (°C)
Northern British Columbia	British Columbia (Canada)	51.944, −122.750	1.7	526	219	−8.2	11.9
Southern British Columbia	British Columbia (Canada)	50.796, −120.439	3.7	481	225	−6.4	14.2
California	California (USA)	41.465, −122.452	11.9	809	98	2.7	22.8

### 2.2 Experimental design

The factorial combination included seeds from three provenances: northern British Columbia (Canada), southern British Columbia (Canada), and California (USA), two competition treatments (grown alone and grown with a neighbor from the same or another provenance), two water treatments (high watering and low watering), and two temperature treatments that averaged 30°C and 20°C. Temperatures were based on previous work done on spring wheat, which tested optimum temperatures (25/15°C daytime and nighttime) and high heat (35/25°C daytime and nighttime). The high‐heat treatment simulates peak summer temperatures at the warmest site (Wang et al., 2016). The 24 treatment combinations were replicated 10 times and arranged in a randomized block design, for a total of 360 pots. During seedling establishment, daytime (6:00–20:00 h) conditions were maintained at 21°C and ambient humidity (40%–50%) and nighttime (20:00–6:00 h) conditions at 15°C and ambient humidity (40%–50%). Furthermore, during daytime conditions, additional lighting was provided with 1000 W halogen sulfide lamps.

Approximately 300 seeds (100 per population) were cold stratified for 8 weeks and germinated on Whatman's ashless filter paper saturated with distilled water in Petri dishes. Seeds were checked daily, and distilled water was added as needed to ensure that germinating seeds did not dry out. From January 20, 2015, to January 22, 2015, germinated seeds which had reached a radicle length of 3 cm were transplanted into pots (10.4 cm × 10.4 cm opening, 5.8 × 5.8 cm base, and 12.9 cm tall) filled with clean fine‐textured sand and saturated with 350 mL of Rorison's solution (after Fraser & Grime, 1998). After 1 week of growing, dead seedlings were replaced with seedlings of the same age. For the first 21 days of growing, each pot received a top watering of 50 mL of Rorison's solution and a bottom watering of distilled water as needed to maintain standing water level of 5 mm. On the 22nd day, temperature and watering treatments began for each of the planting combinations. Pots were evenly and randomly split among two greenhouse compartments. One compartment averaged 20°C (24 h) and 45% relative humidity, and the other averaged 30°C (24 h) and 45% relative humidity. Every 5 days, each pot received a top watering of 350 mL of Rorison's solution. To produce the high‐watering treatment, a subset of pots received an additional bottom watering of 5 mm of distilled water. Before receiving water, one random pot from a low‐watering treatment and one random pot from a high‐watering treatment in each replicate were measured for moisture content using an Extech M0750 soil moisture probe. Any algae found growing in the pots was gently scraped off with a scoopula. Pots which had noticeable algae contamination were documented and excluded from analysis. After 90 days, all plants were measured for height and clipped to soil level. Collected shoot biomass was dried at 60°C for 48 h and weighed. Analysis was conducted with only shoot biomass as roots of competing plants were difficult to distinguish from one another.

### 2.3 Statistical analysis

We tested the response of plant shoot biomass of target plants to watering, temperature, and provenance, when planted alone and under intraspecific competition with other individuals. For each of the competition experimental pots, one plant was designated as the target plant and the other as the competitor. Competition strength was assessed using relative interaction intensity: $\;\mathrm{RII}=\frac{P_{\mathrm{mix}}-P_{\text{control}}}{P_{\mathrm{mix}}+P_{\text{control}}}$, where $P_{\mathrm{mix}}$ is the shoot biomass of a plant growing with a competitor, while $P_{\text{control}}$ is the biomass of a plant grown alone. The RII has limits of −1 (complete competition) to +1 (complete facilitation) (Armas et al., 2004). Three (one for target plants from each provenance) three‐way analyses of variance (ANOVAs) were used to examine the effects of competition, temperature, and water input on biomass of bluebunch wheatgrass from each of northern British Columbia (Canada), southern British Columbia (Canada), and California (USA). Three three‐way ANOVAs were also used to test the effects of competitor provenance, temperature, and watering treatments on shoot RII of target plants from each provenance. For each pot, we included both plants as target and competitor and included pot as a random factor to account for any correlation. For all analyses, significant main or interaction effects were followed by post‐hoc tests, adjusting for multiple comparisons. Analyses were conducted using R software version 3.5.2 (R Core Team, 2019).

## 3 RESULTS

### 3.1 The effects of water input and temperature on shoot biomass of *Pseudoroegneria spicata* plants

**FIGURE 1:**
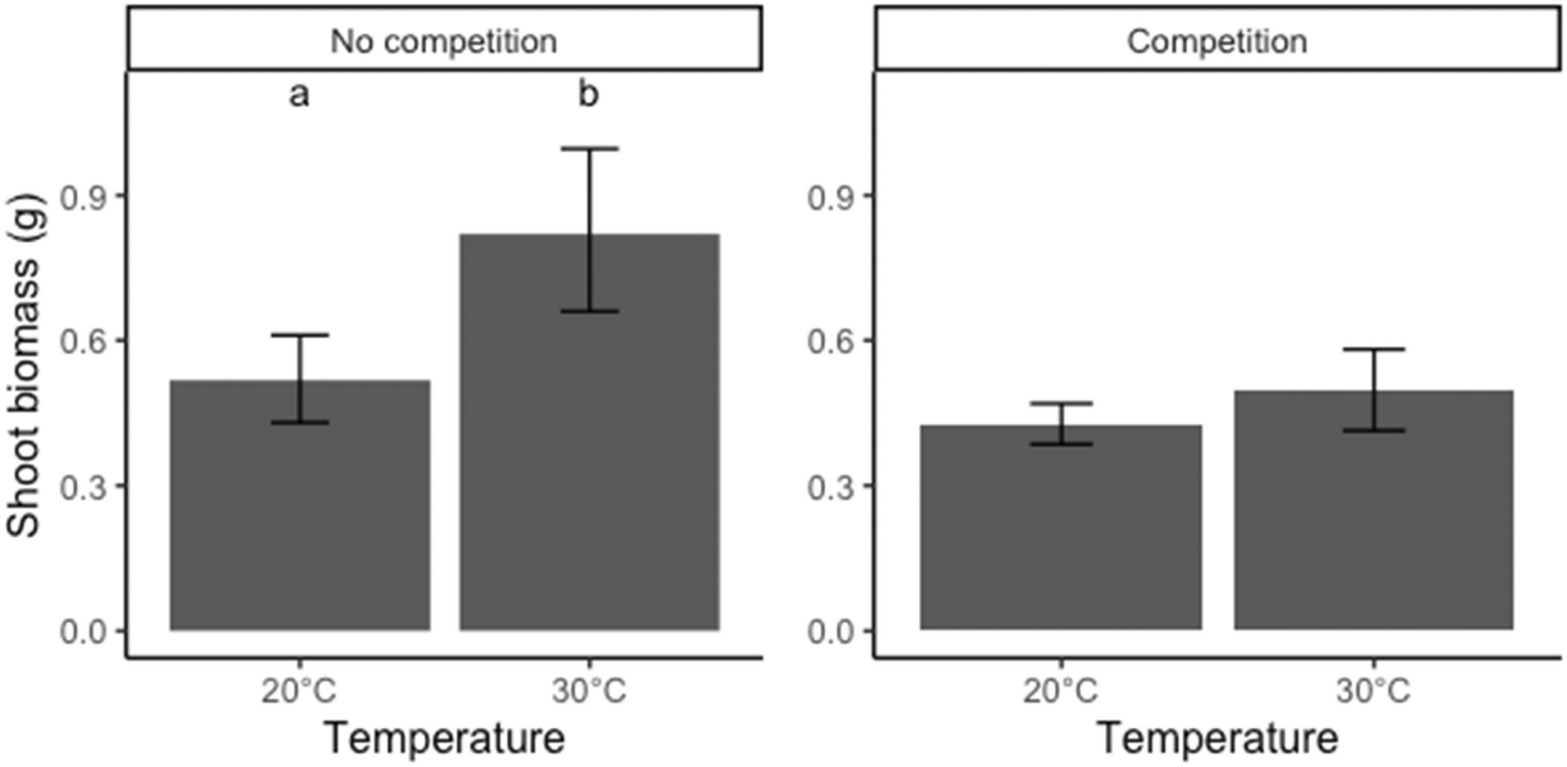
Shoot biomass (showing means and 95% confidence intervals) of *Pseudoroegneria spicata* plants grown at different temperatures, alone and in pairwise competition. Bars with different letters indicate significant differences at *p* < .05.

**FIGURE 2:**
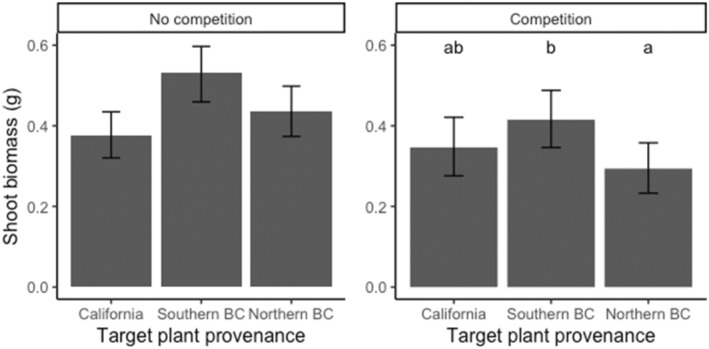
Shoot biomass (showing means and 95% confidence intervals) of *Pseudoroegneria spicata* plants grown from seeds obtained from California, southern British Columbia, and northern British Columbia in western North America and grown in pairwise competition (right) and alone (left). Bars with different letters indicate significant differences at *p* < .05.

In the absence of competition, plants grown at 30°C had significantly higher shoot biomass compared to those grown at 20°C, but there was no difference between temperature treatments under competition (Table 2, Figure 1). We also found no significant differences in shoot biomass between plants grown from seeds obtained from the three provenances when there was no competition. Under pairwise competition, plants grown from seeds obtained from southern British Columbia produced the highest shoot biomass, while those from northern British Columbia produced the lowest shoot biomass (Table 2, Figure 2).

**TABLE 2:** Summary of three‐way ANOVAs testing the shoot biomass of plants obtained from *Pseudoroegneria spicata* plants obtained from northern British Columbia and southern British Columbia in British Columbia and California to water and temperature treatments, alone (no competition) and in pairs (competition).

	NumDF	No competition			Competition		
		DenDF	*F* value	*p* Value	DenDF	*F* value	*p* Value
Location	2	70	2.830	.066	**196.529**	**4.719**	**<.001**
Water	1	70	1.248	.268	97.455	0.652	.422
Temperature	**1**	70	**10.906**	**.002**	97.455	0.002	.964
Location × Water	2	70	0.006	.994	196.529	1.728	.180
Location × Temperature	2	70	0.662	.519	196.529	1.994	.139
Temperature × Water	1	70	0.630	.430	97.455	0.025	.874
Location × Water × Temperature	2	70	1.492	.232	196.529	0.646	.525

*Note*: Values in bold are significant at *p* < .05.

### 3.2 The effects of water input, temperature, and competitor provenance on intraspecific competition in *Pseudoroegneria spicata*

**FIGURE 3:**
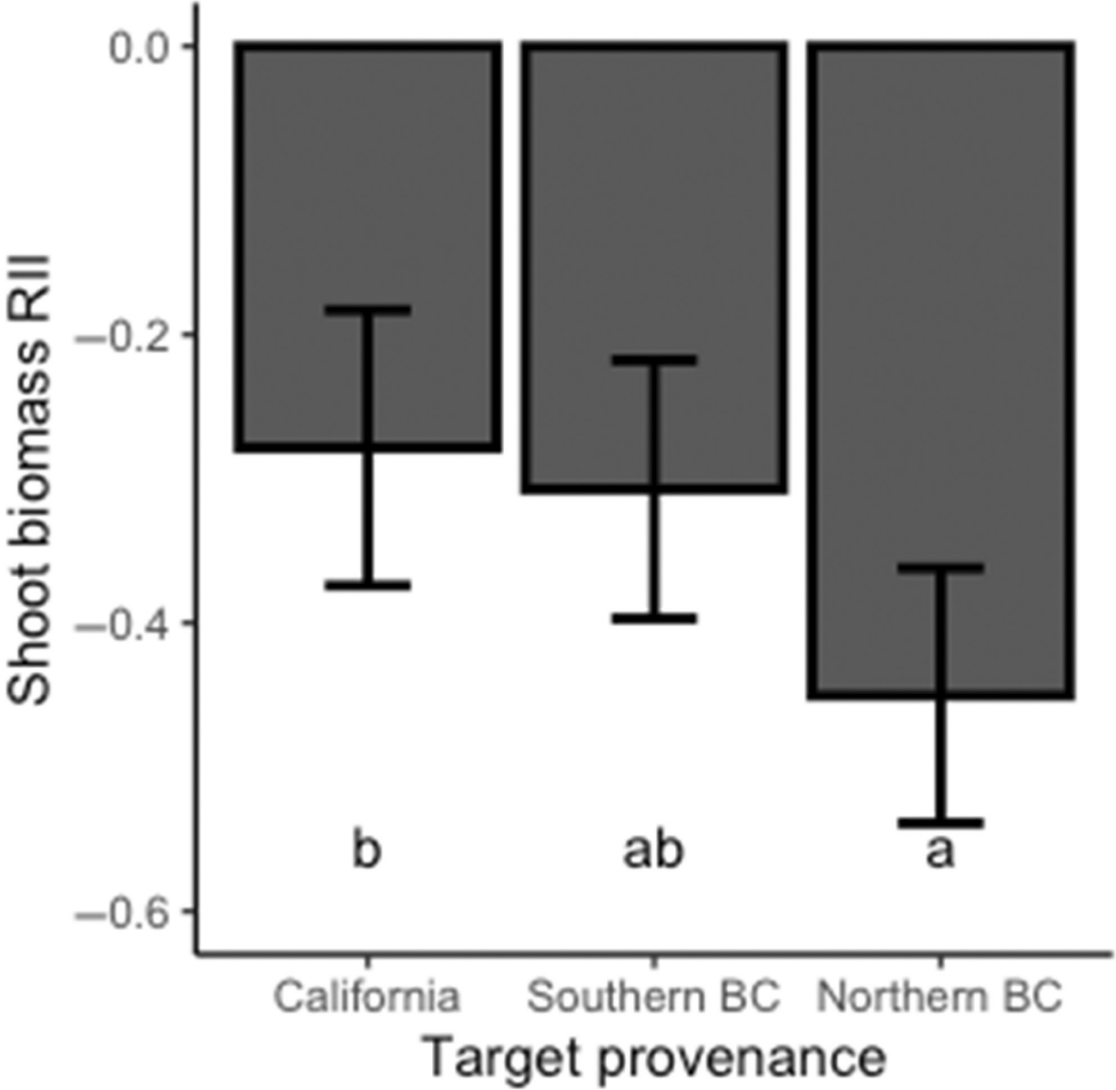
The relative interaction intensity (showing means and 95% confidence intervals) of *Pseudoroegneria spicata* target plants grown from seeds obtained from California, southern British Columbia, and northern British Columbia in western North America. Bars with different letters indicate significant differences at *p* < .05.

**FIGURE 4:**
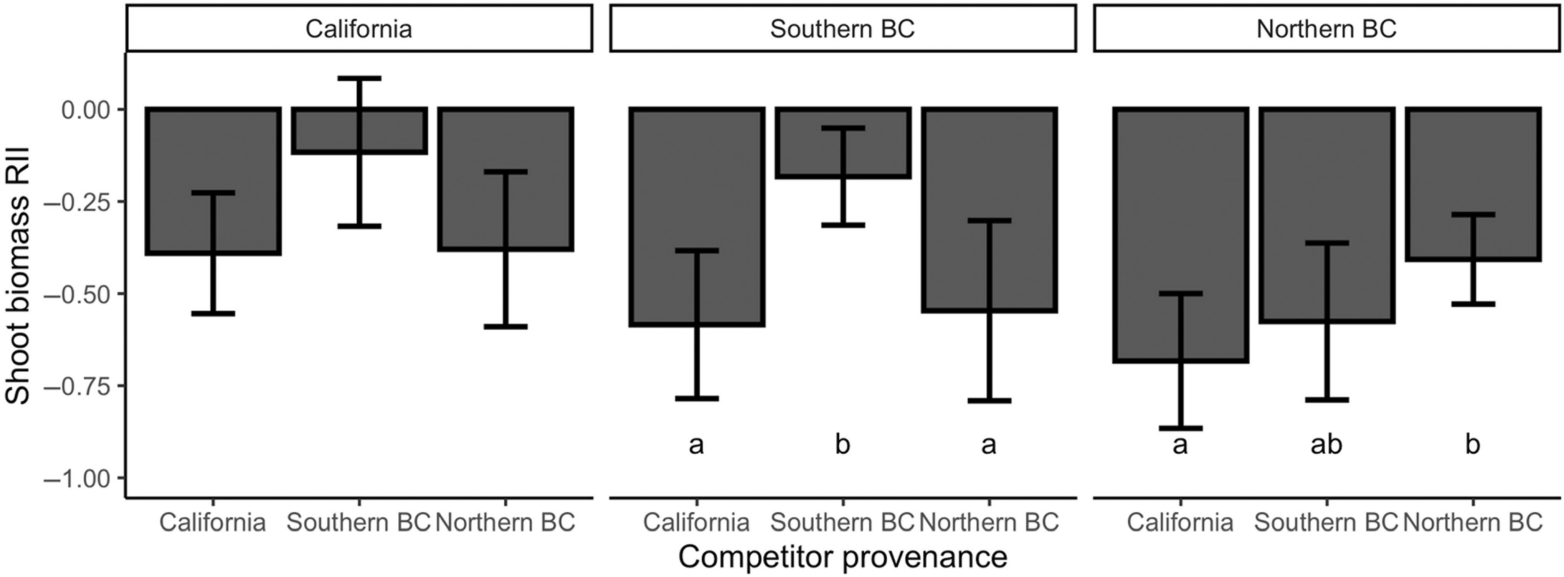
The relative interaction intensity (showing means and 95% confidence intervals) of the competitive effects of *Pseudoroegneria spicata* competitor plants obtained from California, southern British Columbia, and northern British Columbia on target plants from all three locations. Bars with different letters indicate significant differences at *p* < .05.

**FIGURE 5:**
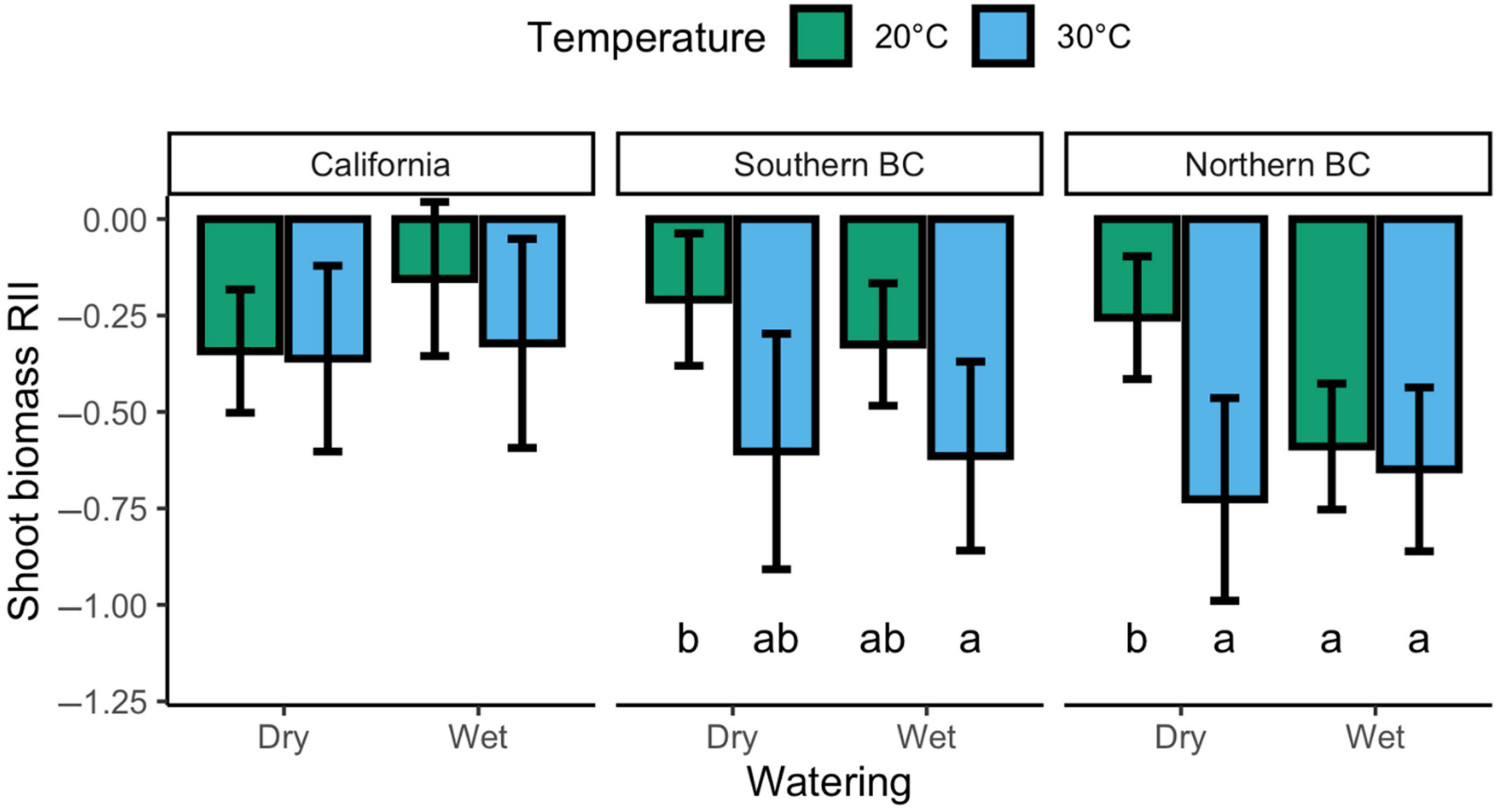
The relative interaction intensity (showing means and 95% confidence intervals) of the competitive effects of *Pseudoroegneria spicata* competitor plants on *Pseudoroegneria spicata* target plants from all three locations, at different temperatures and water inputs. Bars with different letters indicate significant differences at *p* < .05.

Overall shoot biomass RII of target plants differed according to location from which the target plant seeds were collected (*F*_2,221.44_ = 4.295, *p* = .015). Target *Pseudoroegneria spicata* plants from California were least suppressed by competitors, while plants from northern British Columbia were the most suppressed (Figure 3). We examined the role of competitor provenance on the strength of competition and found that northern British Columbia target plants were more suppressed by competitor plants from California, compared to competitors from the same provenance (Table 3, Figure 4). Target plants from southern British Columbia were more strongly suppressed when competing with plants from other locations, compared to when competing with plants from southern British Columbia (Table 3, Figure 4). In addition, target plants from northern British Columbia were least affected by competition under 20°C and dry watering conditions (Table 3, Figure 5). Target plants from southern British Columbia were also more strongly suppressed at 30°C with higher moisture conditions than at 20°C with low moisture (Table 3, Figure 5). RII of plants from California did not differ due to water input or temperature (Table 3, Figure 5).

**TABLE 3:** Three‐way ANOVA table testing the relationships between water, temperature, and competitor identity on relative interaction intensity (RII) calculated using shoot biomass of *Pseudoroegneria spicata* obtained from northern British Columbia and southern British Columbia in British Columbia and California.

	NumDF	Northern British Columbia			Southern British Columbia			California		
		DenDF	*F* value	*p* Value	DenDF	*F* value	*p* Value	DenDF	*F* value	*p* Value
Competitor identity	2	**51.479**	**3.456**	**.039**	**46.158**	**7.393**	**.002**	37.309	2.609	.087
Temperature	1	**52.980**	**6.810**	**.012**	**52.193**	**8.984**	**.004**	41.217	0.716	.402
Watering	1	52.980	1.591	.213	52.193	0.317	.576	41.217	1.060	.309
Competitor identity × Temperature	2	51.479	1.389	.258	46.158	1.128	.332	37.309	1.433	.252
Competitor identity × Watering	2	51.479	1.763	.182	46.158	1.004	.374	37.309	0.644	.531
Temperature × Watering	1	**52.980**	**4.110**	**.048**	52.193	0.210	.648	41.217	0.449	.507
Competitor identity × Temperature × Watering	2	51.479	0.054	.947	46.158	0.577	.566	37.309	0.143	.867

*Note*: Values in bold are significant at *p* < .05.

## 4 DISCUSSION

The success of grassland restoration efforts may be improved by matching the environmental conditions in the plant provenance to that of the restoration location. In a greenhouse, we assessed the intraspecific competitive response of a native grass obtained from three provenances in western North America, to differences in water input and temperature. Our results show within‐species differences in *Pseudoroegneria spicata*, with more intense competitive interactions in target plants obtained from Northern, colder locations. Inter‐provenance competitive interactions were also more intense than intra‐provenance interactions, at warmer temperatures.

Higher temperatures are expected to lead to increased productivity (Baldwin et al., 2014; Dawes et al., 2015), which occurred in the absence of competition. We also found that plants from southern British Columbia had higher shoot biomass in the presence of competition than those from the colder northern British Columbia. Seeds from warmer locations are likely adapted to higher temperatures and longer growing seasons (Sniderhan et al., 2018). This should allow these plants grow more quickly under high‐resource conditions, compared to plants obtained from colder locations, which conserve resources to survive extreme conditions (D'Hertefeldt et al., 2014; Sniderhan et al., 2018). This result also suggests that phenotypic plasticity for shoot biomass in response to temperature may be present in *Pseudoroegneria spicata*, although biotic conditions may influence plastic responses.

We found that target plants from northern British Columbia were more strongly suppressed than those from California. In addition, competitive responses were least negative in intra‐provenance competition in plants from northern British Columbia and southern British Columbia, suggesting that bluebunch wheatgrass is likely less inhibited when competing with conspecifics from the same location. Competitive interactions intensified with increasing distance between target plant provenance and competitor plant provenance. Gustafson et al. (2004) found that local and non‐local populations of *Andropogon gerardii* displayed differences in competitive ability with local target plants performing better against non‐local competitor plants. Similarly, Huang and Peng (2016) showed that the strength of intraspecific competition in an invasive plant became more intense when target and competitor plants from more distant locations were paired with each other. However, other studies have documented a lack of difference in competitive ability in native forbs from different locations in Europe (Bischoff et al., 2006, 2010), in contrast to this study. The difference in competitive abilities in plants used in our study may arise as plants from different provenances are less likely to encounter one another, leading to selection for less intense competition (Zhang, Zhou, et al., 2019).

Another important finding was that the intensity of competition was stronger at higher temperatures and high‐water inputs in northern British Columbia and southern British Columbia, compared to lower temperatures and low‐water inputs, but did not differ in target plants from California. Our results suggest mixed influence of local adaptation on competitive interactions. Plants from northern British Columbia and southern British Columbia were more weakly competitively suppressed at low temperatures compared to high temperatures, while competition in plants from California was unaffected. Rice and Knapp (2008) found that local adaptation was intensified by competition in native bunchgrasses. It appears that the hot and dry growing season conditions experienced by plants from California allow these plants to display competitive tolerance across our experimental conditions. Our results are supported by a meta‐analysis by Liu et al. (2018) who found that experimental warming increased grass abundance in an alpine grassland. Bontrager and Angert (2019) also showed that introduction of plants from warmer locations resulted in increased adaptive genetic variation in northern ranges. Our experimental results are therefore supported by studies that show that warming under climate change is likely to favor plants from warmer locations. In Liancourt et al. (2013), water availability influenced competitive interactions in a native grass with higher competitive intensity at increased water availability similar to our results for northern British Columbia and southern British Columbia target plants.

Our study only ran for 12 weeks; therefore, our conclusions are limited to early‐stage establishment interactions between species. However, we show that response to competition can be dependent on the source of propagules. When considering sources of grass propagules for ecological restoration or assisted migration due to species loss, transplanting propagules from southern locations to northern locations is recommended based on our study. While controlled experiments have higher rates of plant survival, they do not mimic environmental conditions as closely as field experiments. Therefore, a natural next step would be to conduct reciprocal transplant experiments between various locations in the field. We only studied intraspecific competition, but the relative importance of intraspecific versus interspecific competition can change with abiotic stress (Adler et al., 2018; García‐Cervigón et al., 2013); therefore, interspecific competition may also be important in determining the response of *Pseudoroegneria spicata* to climate change.

## AUTHOR CONTRIBUTIONS

**Sabina Donnelly:** Conceptualization (equal); data curation (equal); formal analysis (equal); funding acquisition (equal); investigation (equal); methodology (equal); resources (equal); writing – original draft (equal). **Morodoluwa Akin‐Fajiye:** Formal analysis (equal); methodology (equal); software (equal); validation (equal); visualization (equal); writing – original draft (equal); writing – review and editing (equal). **Lauchlan Fraser:** Conceptualization (equal); funding acquisition (equal); investigation (equal); methodology (equal); project administration (equal); supervision (equal); writing – review and editing (equal).

## Data Availability

Data used for this paper is available on Figshare: https://doi.org/10.6084/m9.figshare.23992956.
